# The “Tail Sign” in Intramuscular Schwannoma

**DOI:** 10.5334/jbsr.2157

**Published:** 2020-08-03

**Authors:** Hamza Khamlichi, Patrick Mailleux

**Affiliations:** 1 Clinique Saint-Luc Bouge, BE

**Keywords:** US, CT, MRI, schwannoma, split-fat, tail

## Abstract

**Teaching point:** This case emphasizes the importance of the “tail sign” to sort the differential diagnosis of soft tissue tumors by highly suggesting schwannoma.

## Case

**Figure 1:**
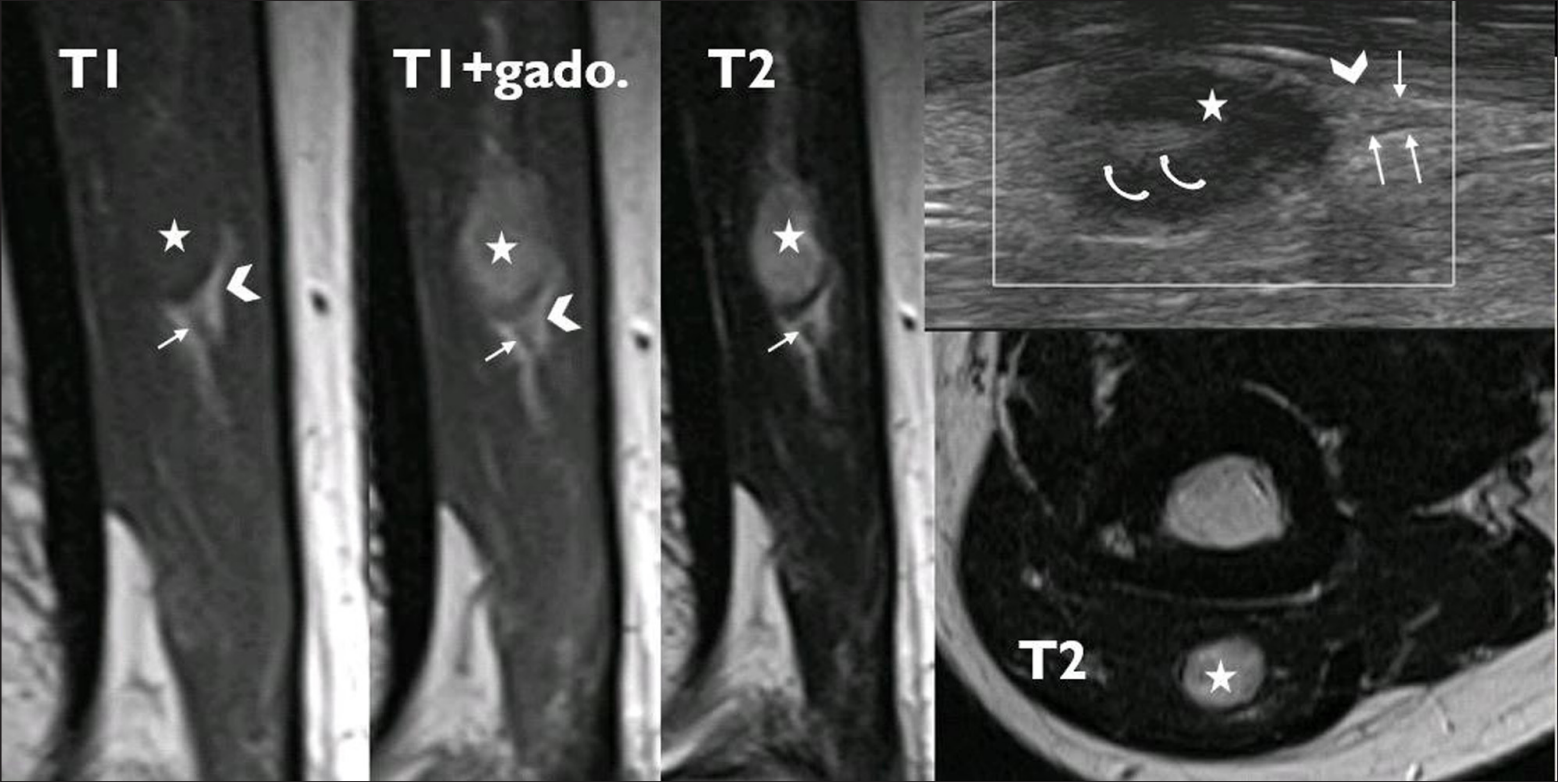


A 50-year-old patient presented with a lump in the distal posterior arm, painful upon compression. Ultrasound showed an intramuscular fusiform mass of 9 × 12 mm in the distal triceps muscle. The tumor (star in Figure 1) is oval in the muscle long axis, hypoechogenic at the periphery, more echogenic centrally (curved arrow in Figure 1). On magnetic resonance (MR), the lesion is isointense on T1-weighted imaging with homogeneous contrast enhancement. It is hyperintense on T2-weighted imaging with a low signal margin. The presence of fat at the poles of the mass is shown (arrowheads in Figure 1). Direct and central continuity with a small nerve branch, the so-called “tail sign”, can be seen (straight arrows in Figure 1) on both ultrasound (US) and MR imaging. The diagnosis of a small benign schwannoma was confirmed at surgery, which lead to complete resection and uneventful recovery.

## Comment

Schwannomas most commonly affect patients aged between 20 and 40 years. They constitute about 5% of benign soft-tissue neoplasms, with the intramuscular variety only representing 2% of schwannomas. Due to the low frequency of this tumor type and the lack of specific signs and symptoms, pre-surgical diagnosis is difficult, especially in small lesions. But, when it is possible to depict the relation of a mass with entering and exiting nerve (the tail sign), hypothesis of a neurogenic tumor should be contemplated. The “split fat” sign that refers to the presence of fat at the upper and lower poles of a lesion as shown in this case (arrowheads in Figure 1) is suggestive of the intermuscular location of the lesion and frequent in benign peripheral nerve sheath tumor, but is not specific [1].

This case emphasizes the usefulness of the “tail sign” on all imaging modalities to sort the differential diagnosis of soft-tissue masses by suggesting peripheral nerve tumor.
